# 100 ps time-resolved solution scattering utilizing a wide-bandwidth X-ray beam from multilayer optics

**DOI:** 10.1107/S0909049509005986

**Published:** 2009-03-20

**Authors:** K. Ichiyanagi, T. Sato, S. Nozawa, K. H. Kim, J. H. Lee, J. Choi, A. Tomita, H. Ichikawa, S. Adachi, H. Ihee, S. Koshihara

**Affiliations:** a Non-Equilibrium Dynamics Project, ERATO, Japan Science and Technology Agency, 1-1 Oho, Tsukuba, Ibaraki 305-0801, Japan; b Tokyo Institute of Technology, 2-12-1 Oh-okayama, Megro-ku, Tokyo 152-8551, Japan; c Center for Time-Resolved Diffraction, Department of Chemistry, KAIST, Daejeon 305-701, Republic of Korea; d High Energy Accelerator Research Organization, 1-1 Oho, Tsukuba, Ibaraki 305-0801, Japan

**Keywords:** time-resolved solution scattering, photodissociation reaction, liquidography, multilayers

## Abstract

100 ps time-resolved X-ray solution-scattering capabilities have been developed using multilayer optics at the beamline NW14A, Photon Factory Advanced Ring, KEK. X-ray pulses with an energy bandwidth of Δ*E*/*E* = 1–5% are generated by reflecting X-ray pulses (Δ*E*/*E* = 15%) through multilayer optics, made of W/B_4_C or depth-graded Ru/C on silicon substrate. This tailor-made wide-bandwidth X-ray pulse provides high-quality solution-scattering data for obtaining photo-induced molecular reaction dynamics. The time-resolved solution scattering of CH_2_I_2_ in methanol is demonstrated as a typical example.

## Abstract 2

A new method of time-resolved solution scattering utilizing X-ray multilayer optics is presented.

## 1. Introduction

Studying photo-induced reactions in the solution phase with subnanosecond time-resolution offers opportunities for understanding fundamental molecular reaction dynamics in chemistry and biology. Time-resolved X-ray diffraction using 100 ps X-ray pulses from a synchrotron source can elucidate the molecular geometry involved in photo-induced reaction pathways (Plech *et al.*, 2004 ▶; Ihee *et al.*, 2005 ▶; Georgiou *et al.*, 2006 ▶; Davidsson *et al.*, 2005 ▶; Kim *et al.*, 2006 ▶; Lee *et al.*, 2006 ▶, 2008*a* ▶,*b* ▶; Kong *et al.*, 2007 ▶, 2008 ▶). An X-ray pulse with ∼3% energy bandwidth has been used for solution-scattering experiments at the ID09B beamline of the European Synchrotron Radiation Facility (Plech *et al.*, 2002 ▶, 2004 ▶; Wulff *et al.*, 2004 ▶, 2006 ▶; Mirloup *et al.*, 2004 ▶; Ihee, 2009 ▶). Significant improvements in the signal-to-noise ratios of the experimental data have been reported for photochemical reactions of halogen compounds in solution. For example, the structural dynamics of C_2_H_4_I_2_ in methanol were studied using the high-flux X-ray pulse at the ID09B beamline (Ihee *et al.*, 2005 ▶), and the reaction pathways and associated transient molecular structures in solution were resolved by the combination of theoretical calculations and global fitting analysis (Lee *et al.*, 2006 ▶; Cammarata *et al.*, 2006 ▶).

**Figure 1:**
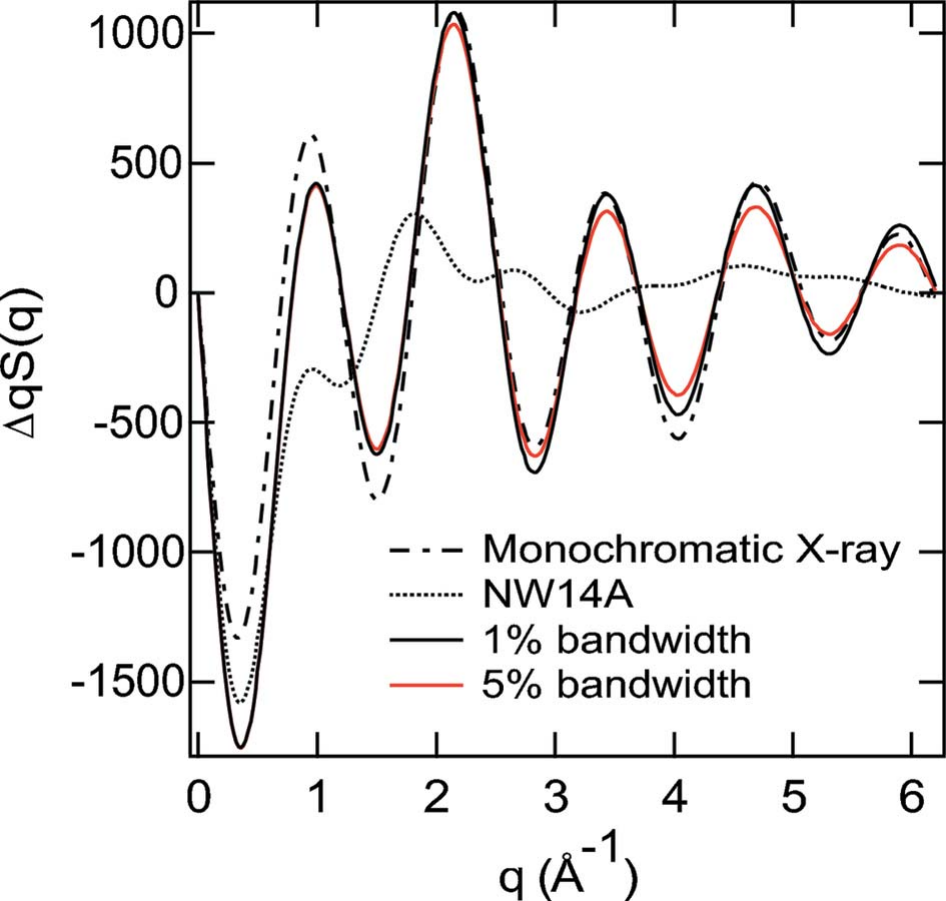
Debye scattering curves calculated for the model reaction C_2_H_4_I_2_ → C_2_H_4_I + I using a 0.01% (monochromatic) Gaussian X-ray energy profile (dot-dashed line), 5% Gaussian X-ray energy profile (red line), 1% Gaussian X-ray energy profile (solid line), and 15% default X-ray energy profile with a long tail (dotted line).

Recently, beamline NW14A at PF-AR, KEK, was constructed as a 100 ps time-resolved X-ray beamline (Nozawa *et al.*, 2007 ▶) using monochromatic or white X-rays. Its high-flux white X-rays have Δ*E*/*E* ≃ 15% energy bandwidth when an undulator of period length 20 mm is used. To check the feasibility of time-resolved scattering with such a wide bandwidth and to search for the optimal bandwidth, we simulated the Debye scattering curves for the reaction C_2_H_4_I_2_ → C_2_H_4_I + I using (i) a 15% bandwidth with the default X-ray energy distribution for the undulator spectrum on NW14A, (ii) a Gaussian spectrum with 5% energy bandwidth, (iii) a Gaussian spectrum with a 1% energy bandwidth, and (iv) a Gaussian spectrum with 0.01% energy bandwidth, as shown in Fig. 1 ▶. The photon flux of the X-ray pulse increases with the energy bandwidth, but the simulation shows that the 15% energy bandwidth with the default spectrum with a long tail is not suitable for time-resolved solution-scattering experiments owing to insufficient *q*-resolution. The long tail of the default X-ray spectrum induces a much higher extent of blurring at high scattering angles than a symmetric Gaussian spectrum with the same bandwidth. For this reason, the X-ray spectrum with a long tail at ID09B of ESRF with ∼3% bandwidth is comparable with a Gaussian spectrum with ∼10% bandwidth. In contrast, when we compare the calculated scattering curve using the Gaussian spectrum with 1% and 5% energy bandwidth X-rays with that with a 0.01% energy bandwidth, three calculated curves seem to reproduce the same quality. In addition, the total flux of the 5% energy bandwidth X-ray beam will be higher than that of the monochromatic X-rays (∼0.01% energy bandwidth) from a Si single crystal by a factor of 250. The total flux of the 5% energy bandwidth X-rays is about five times more than that of the 1% energy bandwidth X-rays. Therefore, the data collecting time using the 5% energy bandwidth X-rays becomes shorter than when using the monochromatic X-rays and the 1% energy bandwidth X-rays. These estimations clearly indicate that the preparation of X-ray pulses with Δ*E*/*E* ≃ 5% has a very significant merit for promoting a time-resolved X-ray solution-scattering experiment, and, thus, prompted us to reduced the bandwidth from the default 15% down to less than the ∼5% energy bandwidth of multilayer optics.

In our experimental set-up, the multilayer optics can produce X-rays with a 1–5% energy bandwidth, and allow us to measure the time-resolved solution-scattering with the undulator at the NW14A beamline. The purpose of this paper is to present a detailed account of achievements with the multilayer optics. We succeeded in collecting high-quality time-resolved solution-scattering data for the CH_2_I_2_ photochemical reaction in methanol and briefly report the experimental aspects.

## 2. Experimental set-up

**Figure 2:**
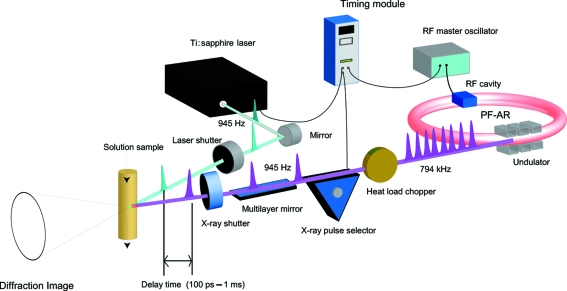
Schematic diagram of the time-resolved solution X-ray scattering at beamline NW14A, PF-AR. The wide-bandwidth (Δ*E*/*E* = 1–5%) X-ray pulses at 945 Hz are provided from the multilayer optics downstream of the X-ray pulse selector. The laser and the X-ray pulse selector are synchronized by using the RF master oscillator.

A schematic diagram of the experimental set-up is shown in Fig. 2 ▶. The experimental system consists of an amplified Ti:sapphire laser system for providing laser pulses to excite the liquid sample, an X-ray pulse selector (XPS) to select single X-ray pulses, a heat-load chopper (Gembicky *et al.*, 2007 ▶), laser and X-ray shutters, and a sapphire nozzle to provide a stable liquid jet. This beamline gives a white X-ray pulse in the energy range 13–18 keV using an undulator with a period length of 20 mm at a repetition rate of 794 kHz and with a pulse duration of about 100 ps. The scattered images were recorded on an integrating charge-coupled device detector (MarCCD165, MarUSA) of diameter 165 mm. Details of the set-up have been described elsewhere (Nozawa *et al.*, 2007 ▶).

## 3. Production of a wide-bandwidth X-ray beam using multilayer optics

**Figure 3:**
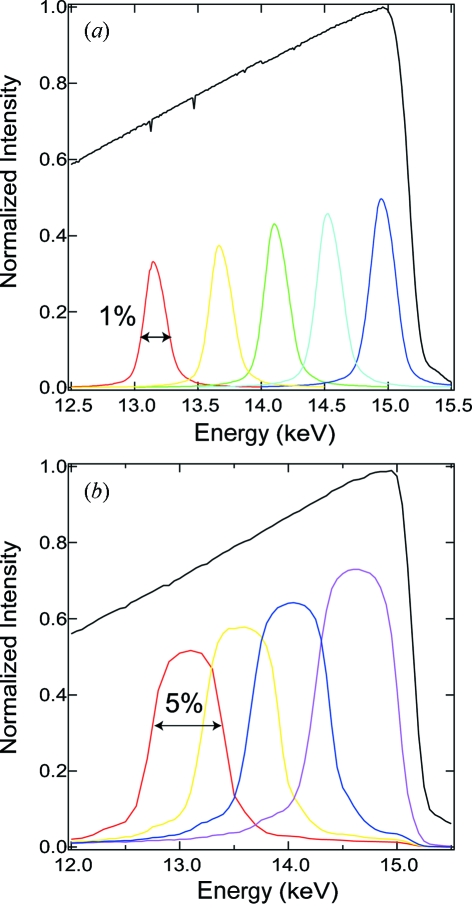
Wide-bandwidth X-ray pulses were produced by multilayer optics from the undulator spectrum. The peak energy position is controlled by changing the incident angle. The black curve is the X-ray spectrum from the undulator, with a gap of 11 mm. (*a*) X-ray spectra using the W/B_4_C multilayer optics. The X-ray bandwidth is about 1%. (*b*) X-ray spectra using the depth-graded Ru/C multilayer optics. The X-ray bandwidth is 5%.

**Figure 4:**
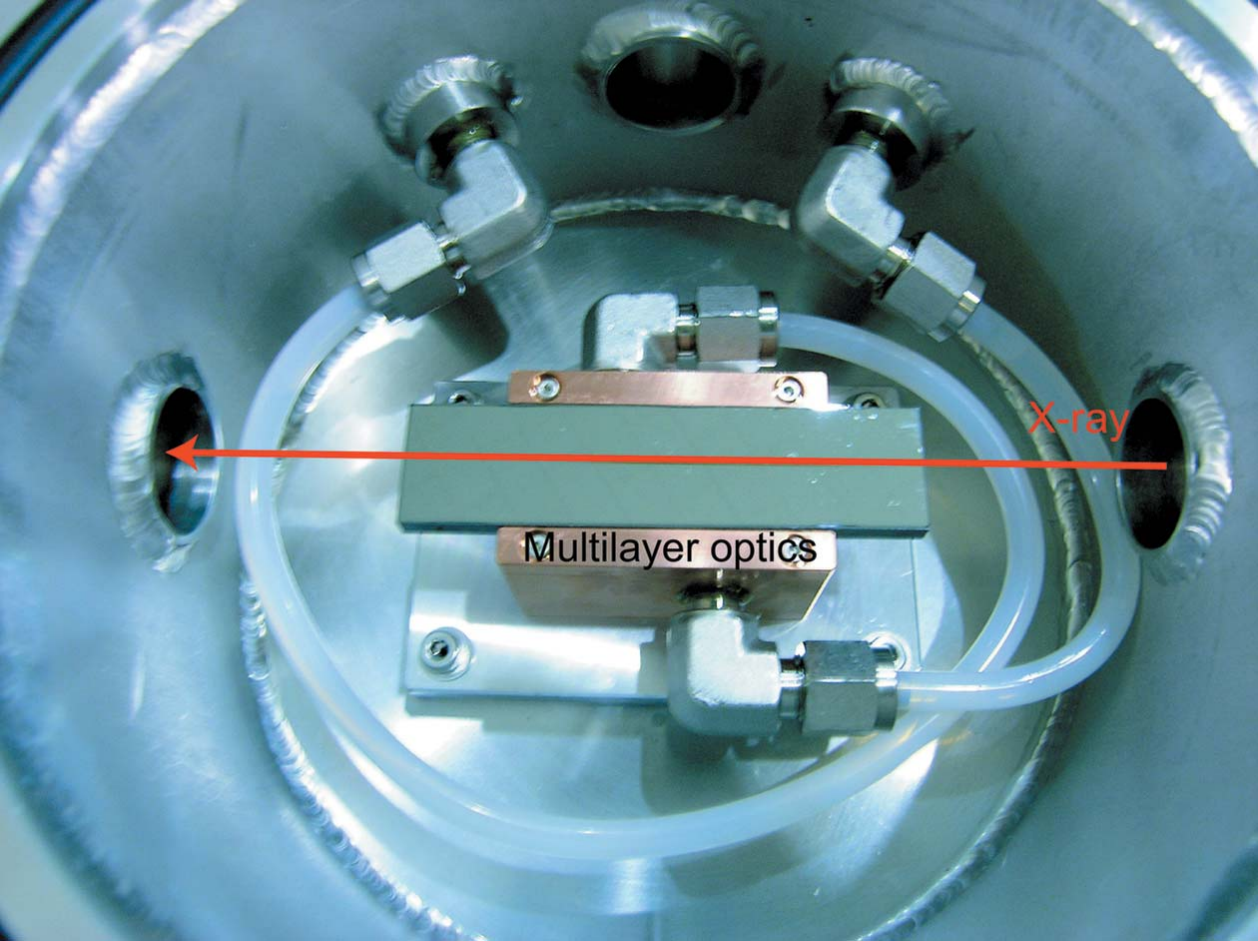
The depth-graded Ru/C multilayer in the vacuum chamber installed at the NW14A beamline at the Photon Factory Advanced Ring at KEK. The multilayer is mounted on a water-cooled holder.

We have utilized two types of multilayer optics. The first one is W/B_4_C (*d* = 27.7 Å, X-ray Company, Russia) on a Si single crystal with a size of 50 × 50 × 5 mm, which provides an X-ray spectrum with ∼1% energy bandwidth and in which the peak energy of the X-ray spectrum can be changed by tilting the angle of the multilayer optics, as shown in Fig. 3(*a*) ▶. The second multilayer, which is a depth-graded Ru/C layer (*d* = 40 Å, NTT Advanced Technology, Japan), produces a ∼5% energy bandwidth from the undulator spectrum, as shown in Fig. 3(*b*) ▶. A real image of the multilayer mirror installed in the vacuum chamber is shown in Fig. 4 ▶. The diameter of the vacuum chamber placed on a swivel stage is 160 mm. The multilayer optics is mounted on a water-cooled copper holder. A white X-ray pulse with a photon flux of 1 × 10^9^ photons per pulse is produced in the energy range at a 1 kHz repetition rate with the XPS. When multilayer optics with 1% and 5% energy bandwidths are used downstream of the XPS, the photon fluxes are 6 × 10^7^ and 3 × 10^8^ photons per pulse, respectively. We can use the discretionary wavelengths and bandwidth in the X-rays for spectra, which is an advantage for the scattering curve corresponding to the asymmetric undulator spectra.

## 4. Time-resolved solution scattering of CH_2_I_2_

**Figure 5:**
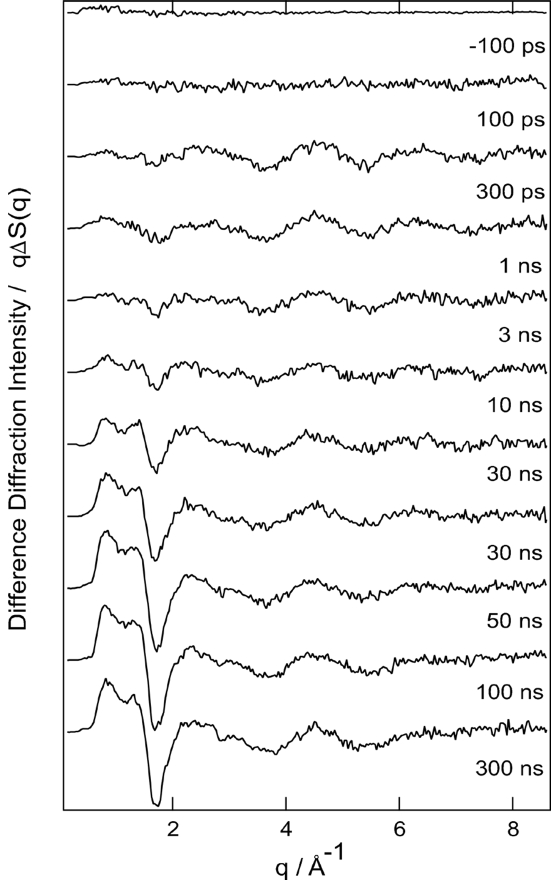
Subnanosecond time-resolved diffraction signal of CH_2_I_2_ in methanol solution as a function of time delay. The differential diffraction intensity was obtained by subtracting the diffraction signal at a reference negative time delay (−3 ns) from the diffraction signal at each time delay.

Photo-induced chemical and biological reactions have been extensively studied by time-resolved spectroscopic techniques and theoretical calculations. Time-resolved X-ray solution scattering makes it possible to probe transient molecular structures in the photo-induced reactions. We measured the time-resolved scattering signals for photodissociation of the iodine atom form CH_2_I_2_ in methanol (Davidsson *et al.*, 2005 ▶). We performed the measurement using X-rays with 5% energy bandwidth at 18 keV to evaluate the feasibility of this set-up. The 60 m*M* CH_2_I_2_ (Aldrich, Japan) in methanol solution was flowed using a liquid jet of thickness 0.3 mm at a flow rate of about 3 m s^−1^. The open jet makes it possible to remove any background signal owing to the scattering of a glass capillary. The CH_2_I_2_ in methanol solution was excited by 267 nm light, the third harmonic of the Ti:sapphire femto­second laser system. To ensure one-photon absorption, the laser pulse width was stretched to ∼2 ps by passing 150 fs laser pulses through a fused silica glass rod cut at the Brewster angle for 267 nm with 175 mm optical length. The spot size of both the X-ray and laser beams on the sample surface was 200 µm diameter. The laser path was set almost parallel to the X-ray path (∼10° tilt), and the intensity of the laser beam on the sample surface was adjusted to ∼35 µJ per pulse. The sample-to-CCD distance and the exposure time were 48.6 mm and 7 s per image, respectively. The CCD detector allowed a 2θ angle range from about 3 to 62° to be measured. Difference diffraction data ware measured at time delays of −200 ps, 100 ps, 300 ps, 1 ns, 3 ns, 10 ns, 30 ns, 50 ns, 100 ns, 300 ns and 1 µs, as shown in Fig. 5 ▶. The CCD images were converted to one-dimensional curves using the *FIT2D* program (http://www.esrf.eu/computing/scientific/FIT2D/). To extract the diffraction intensity change alone, the data for an unperturbed sample at −3 ns were subtracted from the diffraction data collected at other time delays. Photo-induced heating of the solvent is evident in the low *q* region (≤ 2 Å^−1^). The change in the high *q* region indicates the photo-induced structural changes of the CH_2_I_2_ molecule. Details of the data analysis will be reported elsewhere.

## 5. Conclusion

Wide-bandwidth X-ray pulses were generated from depth-graded Ru/C and W/B_4_C multilayer optics for time-resolved X-ray solution scattering. The symmetric shape and the bandwidth (Δ*E*/*E* = 1–5%) of the energy spectra of the X-ray pulse are suitable for time-resolved solution-scattering experiments, and quantitative analysis of photo-induced molecular reaction dynamics in solution. We successfully measured the solution scattering from CH_2_I_2_ in methanol and the time dependence of the difference scattering was presented.
